# Development of immunocompetent full thickness skin tissue constructs to model skin fibrosis for high-throughput drug screening

**DOI:** 10.1088/1758-5090/ad998c

**Published:** 2024-12-13

**Authors:** Yi Wei Lim, Russell Quinn, Kapil Bharti, Marc Ferrer, Hoda Zarkoob, Min Jae Song

**Affiliations:** 1 National Center for Advancing Translational Sciences, National Institutes of Health, Rockville, MD 20850, United States of America; 2 National Eye Institute, National Institutes of Health, Bethesda, MD 20814, United States of America

**Keywords:** skin model, macrophage, immunocompetence, high-throughput, fibrosis

## Abstract

The lack of the immune component in most of the engineered skin models remains a challenge to study the interplay between different immune and non-immune cell types of the skin. Immunocompetent human *in vitro* skin models offer potential advantages in recapitulating *in vivo* like behavior which can serve to accelerate translational research and therapeutics development for skin diseases. Here we describe a three-dimensional human full-thickness skin (FTS) equivalent incorporating polarized M1 and M2 macrophages from human peripheral CD14^+^ monocytes. This macrophage-incorporated FTS model demonstrates discernible immune responses with physiologically relevant cytokine production and macrophage plasticity under homeostatic and lipopolysaccharide stimulation conditions. M2-incorporated FTS recapitulates skin fibrosis phenotypes with transforming growth factor-*β*1 treatment as reflected by significant collagen deposition and myofibroblast expression, demonstrating a M2 potentiation effect. In conclusion, we successfully biofabricated an immunocompetent FTS with functional macrophages in a high-throughput (HT) amenable format. This model is the first step towards a HT-assay platform to develop new therapeutics for skin diseases.

## 1. Introduction

The skin harbors a highly specialized immunological niche crucial for tissue homeostasis, defense against harmful pathogens and modulating tissue repair [1]. The skin immune system is tightly regulated for the proper functioning as a barrier interface, and its dysregulation leads to the manifestation of inflammatory skin diseases [1]. One of the main innate immune cells that make up the immunological niche of the skin are macrophages, predominantly found in the dermis [2]. Macrophages are heterogenous cell populations that exhibit plasticity depending on their microenvironment [3–5]. The complex mechanism of macrophage activation was oversimplified as a polarization of two opposite states, the M1 (classical) and the M2 (alterative) activation state [6]. During tissue injury and infection, M1 macrophages develop in response to interferon *γ* (IFN*γ*) and microbial products such as lipopolysaccharide (LPS). IFN*γ* is upregulated during an infection and is mainly secreted by Th1 and CD8^+^ cytotoxic lymphocytes, natural killer cells and other antigen presenting cells. LPS is the main component of the outer membrane found on gram-negative bacteria and is recognized by cell surface receptor CD14, transmembrane signaling receptor toll-like receptor (TLR)-4 and its accessory protein, myeloid differentiation factor-2 (MD-2) by macrophages and gets activated [7, 8]. Taking advantage of this, LPS stimulation has become a prevalent method for studying pro-inflammatory mechanisms of innate immunity. M1 macrophages are crucial to clear foreign debris, pathogens, and dead cells during an insult and initiate pro-inflammatory responses [9–11]. After pathogen clearance and at later stages of healing, M1 macrophages transition into anti-inflammatory, pro-resolution M2 macrophages. In contrast to M1, M2 macrophages are associated with secretion of anti-inflammatory cytokines and support tissue repair by promoting angiogenesis and tissue remodeling [12]. Cytokines associated with type II immune responses such as interleukin (IL)-4, IL-13 and IL-10 induce a M2 phenotype. IL-4 and IL-13 are mainly produced by Th2, mast cells and basophils, while IL-10 is mainly produced by macrophages, T cells, B cells, mast cells and keratinocytes as part of the homeostatic response to infection and inflammation [10]. The numbers and phenotypes of the different macrophage subsets need to be properly regulated for skin tissue homeostasis.

Acute wounds normally heal in an orderly, well-orchestrated manner to restore the tissue into its original anatomical structure and function [13]. During this process, fibroblast migrates into the wound site and transforms into myofibroblasts, the cells responsible for depositing new extracellular matrix (ECM). These ECM crosslink and organize to return the tissue into homeostasis [13]. Efficient ECM remodeling is balanced by both deposition and degradation involving metalloproteinases (MMPs) and their inhibitors (tissue inhibitors of MMPs, TIMPs) as well as establishing highly organized ECM structures [14]. While this process is integral to tissue recovery, it may also result in tissue fibrosis when it is dysregulated [15]. During normal wound healing process, myofibroblast population should dissipate when the wound is close, via apoptosis or revert into quiescent fibroblast [16, 17]. In fibrotic conditions, myofibroblast apoptosis is delayed and they continue to produce inappropriate amounts of collagen, among other ECM components [18]. This persistent myofibroblast activation ultimately leads to tissue thickening and hardening, which can result in organ malfunction and ultimately death [19]. There are many etiologies of fibrotic disorders owing to their complex pathophysiological mechanisms, and it is accepted that macrophages play key roles in the initiation, progression, and resolution of fibrosis [20]. Increased infiltration and activation of macrophages in scleroderma skin fibrosis biopsies has been previously reported, particularly pro-fibrotic M2 macrophages [21–25]. Macrophages regulate fibrogenesis by secreting factors that recruit and activate fibroblast and other inflammatory cells such as transforming growth factor *β*1 (TGF*β*1), IL-6 and platelet-derived growth factor (PDGF) [20, 26, 27].

Drug development for inflammatory skin diseases has been hindered due to the lack of pathophysiological relevant models. Monocellular 2D culture has proven ineffective to model skin inflammatory disease as they lack multicellular communication and microenvironmental factors found in inflammatory skin diseases. Animal models have been used to analyze irritation from chemical exposure and investigate complex skin diseases but may produce results which are not directly translatable to humans due to differences in genetics, anatomical, physiological, and immunological features. These inaccuracies due to the fundamental interspecies differences lead to insufficient translation between animal models to human patients [28]. Therefore, there is a critical need to develop immunocompetent three-dimensional (3D) skin tissue models to study the interplay between different immune cell subsets and the non-immune compartments in the skin and use as pre-clinical testing assays for the development of treatments for inflammatory skin diseases.

Recent advances have generated 3D engineered skin models with immune cell inclusion to increase physiological complexity of skin disease models. Some of the immunocompetent 3D engineered skin models include integration of Langerhans cells and dermal dendritic cells in full thickness skin (FTS) equivalents to study effects of allergen or irritant exposure as well as ultraviolet radiation on skin [29–31]. Immunocompetent skin models that incorporate macrophages in the dermis to study wound healing and vascularization have also been generated [32–34]. Recently, disease-relevant immune cells such as Th1 and Th17 T cells have been used to model skin psoriasis disease [35, 36]. Scleroderma patient-derived monocytes incorporated into FTS model were shown to recapitulate cutaneous fibrosis phenotype [37]. The main challenge of developing an immunocompetent 3D skin remains the effective inclusion of multiple immune components to mimic native skin [38, 39].

Here we developed an immunocompetent FTS model in a 96-well transwell format as a high-throughput (HT) screening amenable platform by incorporating primary human macrophages derived from peripheral CD14^+^ monocytes. We validated the immunocompetence of macrophage-incorporated FTS (M*ϕ*-FTS) model as well as macrophage plasticity under LPS stimulation. M2-incorporated FTS recapitulates fibrosis phenotype under TGF*β*1 treatment, highlighting that M2 potentiates the fibrosis outcomes.

## 2. Materials and methods

### 2.1. Cell culture and macrophage polarization

Commercially available frozen human peripheral blood CD14^+^ monocytes (STEMCELL Technologies, Vancouver, Canada) were used for macrophage polarization. The commercial CD14^+^ monocytes isolated via negative selection (Cat#200-0167) or positive selection (Cat#70035) were used for the study, and the company stated over 85% of purity of the CD14^+^ cells. Only CD14^+^ monocytes isolated from healthy donors that are HIV-1, HIV-2, Hepatitis B, Hepatitis C and CMV negative were purchased. Both male and female donors were included. Primary CD14^+^ monocytes were polarized into M1 with GMCSF for 5 d and IFN*γ*, IL-6 and GMCSF for an additional 3 d, or M2 with MCSF for 5 d and IL-4, IL-13, IL-6 and MCSF for an additional 3 d in RPMI 1640 Medium, GlutaMAX supplement (ThermoFisher scientific, Waltham, MA USA), containing 10% double heat inactivated fetal bovine serum (FBS, GeminiBio, West Sacramento, CA, USA). All cytokines (R&D systems, Minneapolis, MN USA) are added at 20 ng ml^−1^ concentration. The cells were plated on the flask at 6.7 × 10^4^ cells cm^−2^. To harvest, the adhered macrophages were washed 1× with DPBS and incubated with CTS TrypLE Select Enzyme (ThermoFisher Scientific) for 15–20 min at 37 °C followed by cell scraping. The dissociation process was neutralized with growth medium and centrifuged at 200 × g for 5 min. The cell pellet was resuspended in growth medium and total viable cell counts were determined by Cellometer Auto T4 (Nexcelom Bioscience, Lawrence, MA, USA). Human primary neonatal dermal fibroblast (American Type Culture Collection, Manassas, Virginia, USA) was cultured in FibroLife S2 Fibroblast Medium (LifeLine Cell Technology, Frederick, MD, USA) for expansion. Primary human epidermal neonatal keratinocytes (ScienCell, Carlsbad, CA, USA) were cultured in keratinocyte medium, KM (ScienCell) for expansion in flasks and for keratinocyte seeding and culturing before switching to differentiation media in FTS protocol. The fibroblast and keratinocyte with only less than passage 4 were used. The fibroblast and keratinocytes were dissociated from the sub-culture flasks using 0.05% trypsin-EDTA and neutralized with growth medium containing 10% FBS.

### 2.2. Biofabrication of Mϕ-FTS

3D printed O-rings by stereolithography 3D Printer (Formlabs, MA, USA) were glued using Kwik-Cast Silicone Sealant (World Precision Instruments, Sarasota, FL, USA) on the basal side of 96 well transwell 1 *μ*m PET membrane (Corning, Glendale, AZ, USA). 50 *μ*l of 100 *μ*g ml^−1^ rat tail collagen type 1 in cold DPBS without calcium and magnesium and then washed with the DPBS and left to dry overnight at room temperature (RT). A fibrinogen solution containing 5 mg ml^−1^ fibrinogen from human plasma (Sigma Aldrich, St. Louis, MO, USA) and 25 *μ*g ml^−1^ aprotinin from bovine lung (Sigma Aldrich) was prepared in DPBS and sterile filtered. 0.6 × 10^6^ cells ml^−1^ dermal fibroblasts with or without 0.42 × 10^6^ cells ml^−1^ macrophages were resuspended with the fibrinogen solution. Immediately after mixing with thrombin (Sigma Aldrich) at 1 U ml^−1^ of the final concentration, 50 *μ*l of the gel–cell mixture was pipetted onto the basal side of the transwell to generate the dermis-like structure. The added gel–cell mixture was left for 15 min at RT to promote gelation followed by submersion in the basal medium A (FDMA) according to previously published protocol [40] (supplementary table 1) with 1 U ml^−1^ thrombin for 24 h at 37 °C. At day 3, The keratinocytes were seeded at 2 × 10^5^ cells cm^−2^ on the apical side. On day 5, the apical KM were aspirated and switched to 50 *μ*l of differentiation media to start epidermis differentiation. On day 6, the culture was subjected to air–liquid interface (ALI) by lifting the transwell insert with a 3D printed plate spacer (custom-made with a Formlabs 3D printer) and by removing medium from the apical side. The medium in the basal region was switched from basal medium A to basal medium B (FDMB) [40] and the basal medium B was refreshed every other day during ALI day 0 through day 4 (ALID0–D4) and every three days with a taller plate insert from ALID4–D10.

### 2.3. Induction of skin fibrosis using Mϕ-FTS

FTS with and without M2 macrophages were generated as described above with minor modification where 50 *μ*l of 30 *μ*g ml ^−1^ fibronectin (ThermoFisher Scientific) in deionized water were coated on the apical side of the membrane for 1 h at RT instead of rat tail collagen type I. Basal medium recipe for fibrosis model can be found in supplementary table 2. At ALID4–D10, recombinant TGF*β*1 (R&D systems) were added at 10 ng ml^−1^ to the fibrosis basal media C to induce fibrosis phenotype. At ALID10, the conditioned media were collected for Luminex analysis and the transwell were submerged in 200 *μ*l of 10 *μ*M mCherry CNA-35 [41] (pET28a-mCherry-CNA35 available from Addgene, protein purification conducted by Curia Global) in fibrosis basal media C for 24 h in the incubator to label collagen for high content imaging. After 24 h of live staining, the plate was washed with DPBS twice for 15 min and barrier function were measured with transepithelial electrical resistance (TEER).

### 2.4. TEER assay

TEER measurements were acquired at ALID8 or ALID11 using automated TEER measurement system (World Precision Instrument, USA). The transwell inserts were filled with 80 *μ*l in the apical region and 240 *μ*l DPBS in the basal region. The contribution of the PET membrane was measured and subtracted from the sample values. The final values of TEER in Ω*cm^2^ are obtained from multiplying the electrical resistance by the growth area of 0.143 cm^2^. The samples that passed our quality control with TEER value higher than 500 Ω*cm^2^ were used in this study.

### 2.5. Cell isolation from FTS and Mϕ-FTS

Only the dermis fraction was used to isolate single cells from dermis (72 h timepoint) or FTS with and without macrophages. Using a surgical scalpel, the dermis of the FTS construct is sliced on the PET membrane to create smaller pieces of the tissue. After that, the tissue pieces were transferred into dissociation buffer that consists of 1.5 mg ml^−1^ Gibco collagenase II (ThermoFisher Scientific), 0.5 mg ml^−1^ Roche DNAseI (Millipore Sigma, Burlington, MA, USA) and 0.2 mg ml^−1^ Gibco Dispase II (ThermoFisher Scientific) in DPBS without calcium or magnesium. 3–5 dermis constructs were pooled into one well that contains 500 *μ*l dissociation buffer. The tissues are incubated in dissociation buffer at 37 °C in an incubator for 30 min. After 30 min of incubation, the tissues in dissociation buffer were pipetted up and down to gently dissociate them and pass through a 100 *μ*m cell strainer (Corning) to obtain single cell suspension. The cell suspension is washed once with growth medium and is ready for downstream applications.

### 2.6. Flow cytometry

Single cell suspension was washed with DPBS twice before staining with 1:1000 eBioscience Fixable viability dye (ThermoFisher Scientific) for 10 min at RT. After viability staining, the cells were washed with cell staining buffer (Biolegend, San Diego, CA, USA) once before adding Human TruStain FcX blocking solution (Biolegend) and incubated for 10 min at RT according to manufacturer’s recommendation. Fluorescent conjugated antibodies as well as True-Stain Monocyte Blocker (Biolegend) were added to the cell suspension and incubated on ice in the dark for 20 min. The cell suspension was washed with cell staining buffer twice and analyzed with BD Fortessa Flow Cytometer. Fluorescent conjugated antibodies used in this study includes 1:100 Alexa Fluor^®^ 488-CD206, 1:154 PE-CD206, 1:50 PE/Cyanine7-CD209, 1:50 Alexa Fluor^®^ 700-CD163, 1:100 Alexa Fluor^®^ 647-CD80, 1:154 PerCP/Cyanine5.5-CD86, 1:154 APC-CD86, 1:200 Pacific Blue™-HLA-DR, 1:50 PE-HLA-DR, 1:100 Brilliant Violet 421™-CD45 and 1:100 PerCP/Cyanine5.5-CD11b (Biolegend, catalogue number and antibody clone can be found in supplementary table 3).

### 2.7. Immunofluorescence

Opera Phenix (Perkin Elmer) was used to obtain confocal image of the FTS. For full tissue antibody staining, the tissues were fixed with 4% paraformaldehyde solution in PBS (ThermoFisher Scientific) overnight at 4 °C. After DPBS wash, the samples were permeated with 0.5% Triton X-100 (ThermoFisher Scientific) for 30 min RT on a rocker. After permeabilization, the tissues were blocked with 1% bovine serum albumin (BSA, Sigma), 5% goat serum (ThermoFisher Scientific) and 0.1% Triton X-100 in DPBS for 30 min RT on the rocker. The samples were stained with anti-myofibroblast primary antibody (1:400, Abcam ab20255, supplementary table 3) in 1% BSA, 0.1% Triton X-100 at 4 °C. The samples were washed twice with DPBS on a rocker for 30 min each RT before adding 1:1000 5 mg ml^−1^ DAPI and 1:300 Alexa Fluor^®^ 647 Goat anti-Mouse IgG (Invitrogen A-21236, supplementary table 3) for 3 h on the rocker, in the dark. For image analysis of wells, mean intensity was obtained from maximum projection images using Columbus software (Perkin Elmer).

### 2.8. Histology

FTS tissues were fixed in 4% paraformaldehyde solution in PBS (ThermoFisher Scientific) overnight at 4 °C and then stored in 30% sucrose DPBS solution until ready for cryosectioning. The tissues were cut out of the transwell using a surgical scalpel and embedded in Tissue-Tek O.C.T. (Fisher Scientific) for sectioning. 10 *μ*m thick cryosections were obtained using the CryoStar NX50 (Thermo Scientific) and placed on positively charged slides. Hematoxylin and eosin (H&E) staining were performed on the Thermo Scientific Gemini AS stainer using the predefined H&E staining protocol. Immunofluorescence staining for tissue sections was performed on the Leica Bond RXm using the standard predefined staining protocol with the AKOYA Biosciences Opal 4-Color Automation IHC kit. Primary antibodies used in this study for staining includes anti-filaggrin antibody (1:5000, Abcam Ab221155), anti-cytokeratin 10 antibody (1:2000, Abcam Ab234313), anti-Ki67 antibody (1:1000, Abcam Ab15580). Opal 520 Fluorophore (1:150), Opal 570 Fluorophore (1:150), Opal 650 Fluorophore (1:150) and DAPI (1:1000) were used in this study. Slide images were taken with Leica Aperio Versa 200 at 10X magnification.

### 2.9. Luminex multiplex assay

Conditioned media from culture were collected and freeze at −80 °C until ready for Luminex Multiplex Assay. Luminex assay kit were customized and purchased from Bio-Techne R&D systems. Selected biomarker concentrations in the culture supernatants were determined by Bio-Techne Human Premixed Multi-Analyte Luminex Kit and Luminex FLEXMAP 3D instrument according to manufacturer’s instruction. The obtained data were analyzed by Bio-Plex Manager software. The analyte measured in this study and their corresponding sample dilution factor includes: TNF (1:2), MMP9 (1:2), IL-10 (1:2), CD14 (1:2), CCL22 (1:2), CCL24 (1:2), PDGFcc (1:2), IL-11 (1:2), CCL13 (1:100), IL-6 (1:100), CXCL10 (1:100), IL-8 (1:100), CCL8 (1:100), pro-collagen 1 alpha 1 (1:100), Fibronectin (1:100).

### 2.10. Statistical analysis

The details for *N, p* values and statistical test performed for each experiment are described in the figure legends or main text. The data is expressed as means ± standard deviation. Paired student’s t-test was applied for two-group comparisons and ANOVA was performed for multigroup comparisons. Sidak’s and Tukey’s multiple comparisons test were conducted to assess significant differences among treatment and sample groups. The significance was denoted by ns: not significant, *p < 0.05, **p < 0.01, ***p < 0.001, ****p < 0.0001 or a: *p* < 0.05, b: *p* < 0.01, c: *p* < 0.001. All data were analyzed using GraphPad Prism.

## 3. Results

### 3.1. Generation of immunocompetent FTS equivalent with primary human macrophages

**Figure 1.**
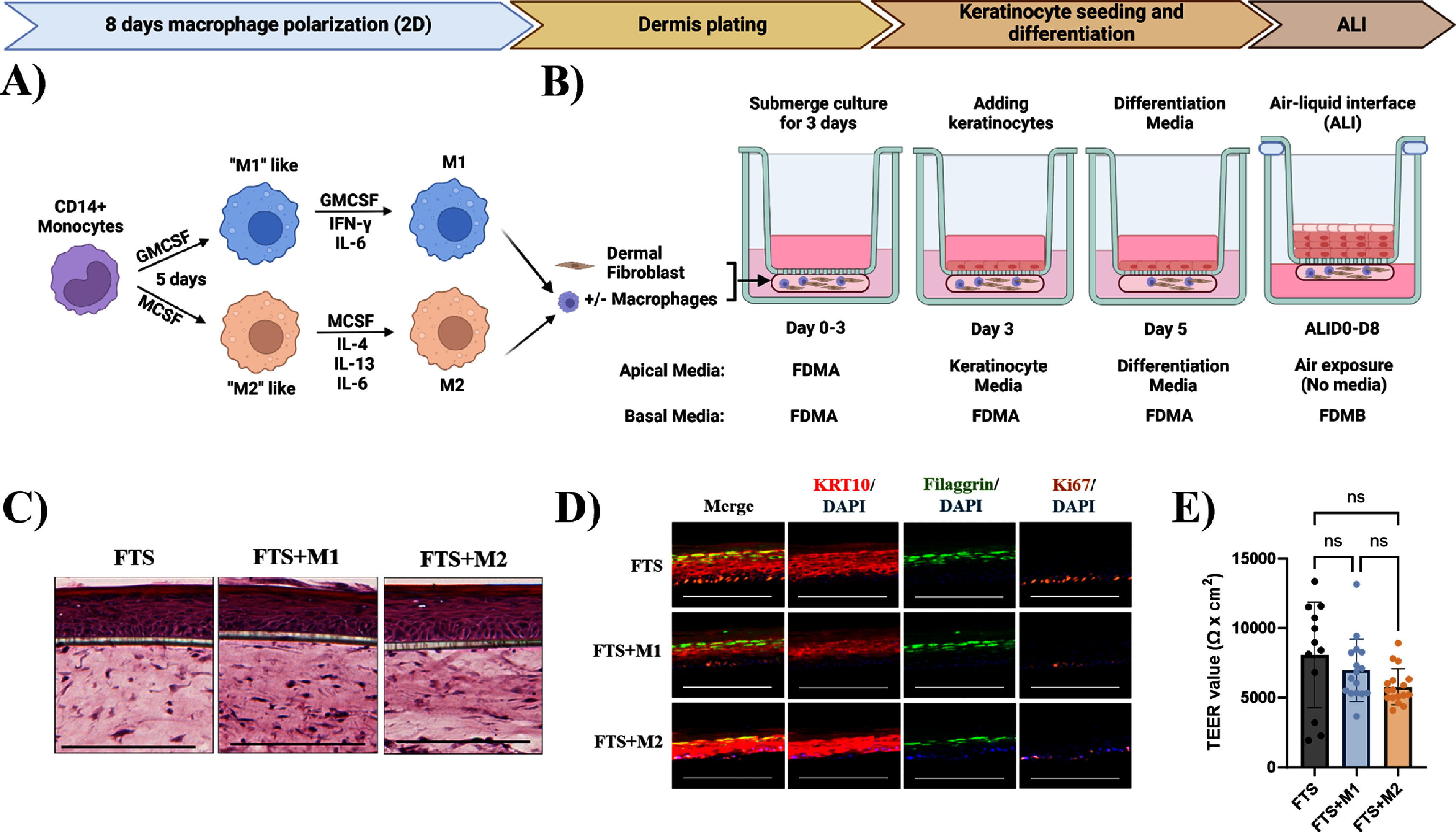
Generation and validation of M*ϕ*-FTS. (A) Schematics of M1 and M2 polarization from CD14^+^ primary human monocytes from peripheral blood. The monocytes were polarized for 8 d prior to harvesting to incorporate into the gel–cell mixture that consists of dermal fibroblast for dermis generation. (B) Schematics of generating FTS in a 96-well transwell plate and the corresponding basal and apical media. Macrophages and dermal fibroblast were plated at the basal side of the transwell at day 0 to generate the dermis, and keratinocytes are seeded at day 3 to the apical side of the transwell membrane. (C) H&E images of FTS with and without macrophages, 10X magnification, scale bar = 200 *μ*m, 10 *μ*m sections, representative of 3 primary monocyte donors. (D) Validation of epidermis differentiation of FTS with and without macrophages using cytokeratin 10 (KRT10), filaggrin, Ki67 and DAPI staining. Representative images from 3 primary monocyte donors, 10X magnification, scale bar = 200 *μ*m, 10 *μ*m sections. (E) TEER measurement on ALID8, *N* = 12–17 per condition pooled from 3 primary monocyte donors, ordinary one-way ANOVA, Tukey’s multiple comparisons test, ns= not significant. (A) and (B) Created in BioRender. Lim [43] BioRender.com/k10f620.

CD14^+^ primary human monocytes were pre-polarized into M1 and M2 macrophages for 8 d based on published protocol with some modifications (figure 1(A)) [42]. We perform routine assessment to ensure the polarization efficiency by measuring expression levels of CD86, CD206 (M1 and M2 markers respectively) and CD11b (pan-myeloid marker) prior to introducing into the FTS model. We modified our previously published protocol for generating FTS model [40] to introduce macrophages. In brief, the fibrin hydrogel consisting of dermal fibroblast with or without the pre-polarized macrophages (figure 1(A)) were pipetted onto the basal side of the transwell membrane. The submerged culture was maintained for 3 d followed by the addition of primary human keratinocytes on the apical side of the transwell membrane. After another 3 d of culture for keratinocyte growth, the culture is brought into ALI for 8 d for keratinocyte differentiation into epidermal layers (figure 1(B)). The incorporation of M1 and M2 macrophages into FTS model did not alter epidermal differentiation and morphology as demonstrated in H&E staining at ALI Day 8 (ALID8, figure 1(C)). Both the early and late differentiation markers of epidermal differentiation, cytokeratin 10 (KRT10) and filaggrin, respectively, were strongly expressed regardless of the macrophage inclusion. In addition, expression of the proliferation marker Ki67 confirmed the characteristics of the epidermal basal layer (figure 1(D)). Furthermore, M1 or M2 incorporation into FTS model did not affect epidermis proliferation as measured by Ki67 quantification of the epidermal basal layer (supplementary figure 1(A)) as well as epidermis thickness measured on H&E-stained tissue section (supplementary figure 1(B)). The M*ϕ*-FTS model demonstrated equivalent barrier functions, as indicated by a TEER measurement of 6985.4 ± 2240.6 Ω*cm^2^ and 5799.4 ± 1292.0 Ω*cm^2^ for FTS + M1 and FTS + M2 respectively (figure 1(E)), demonstrating that macrophage incorporation does not affect epidermis differentiation and barrier function.

We next assessed the viability of incorporated macrophages within our FTS model and compared two timepoints: 72 h post dermis plating and ALI day 8. We also assessed if the presence of other cell types would affect the viability of the incorporated macrophages using two methods of modular study: method 1 and 2) neither fibroblast nor keratinocytes in the macrophage alone condition at 72 h timepoint; method 2) no fibroblast in macrophage and epidermis condition at ALI day 8 timepoint (supplementary figure 2(A)). Dermis + macrophages represent 72 h post-dermis plating samples while FTS + macrophages are ALI Day 8 samples (only ALI day 8 samples contain an epidermis to represent FTS).

We conducted tissue digestion on the dermis to obtain single cell suspension from the two timepoints and stained for CD45 (pan-immune cell marker to exclude fibroblast in analysis), CD11b (pan-myeloid marker) and viability dye and conducted flow analysis to assess the frequency of live^+^CD45^+^CD11b^+^ macrophages. In method 1, we found a significant lower frequency of live^+^CD45^+^CD11b^+^ macrophages at ALI Day 8 compared to 72 h timepoint for all samples except FTS + M1 where there was a decreasing trend. In the absence of dermal fibroblast and keratinocytes, M1 and M2 alone in hydrogel also demonstrate a significant decrease in live^+^CD45^+^CD11b^+^ macrophage frequency at ALI day 8. This data suggests that in the presence or absence of fibroblast and epidermis, there is a decrease in live macrophage frequency by the end of the FTS culture timeline at ALI day 8 (supplementary figure 2(B)).

Next, we investigated the viability of incorporated macrophages within the skin models with and without dermal fibroblast (method 2, supplementary figure 2(A)) by seeding keratinocytes on macrophage alone in hydrogel wells following FTS model protocol. In terms of frequency of live^+^CD45^+^CD11b^+^ macrophages, we observed a decrease in all samples except by FTS + M1 at ALI day 8 compared to 72 h post dermis plating timepoint (supplementary figure 2(C)). We next assessed the relative number of CD45^+^CD11b^+^ macrophages of the two timepoints by multiplying the percentages of CD45^+^CD11b^+^, live cells, single cells and total cells gate to the cell count after tissue digestion. We observed that both in the absence and presence of dermal fibroblast, we saw a decrease in CD45^+^CD11b^+^ cell number at ALI day 8 compared to 72 h timepoint for all samples except in FTS + M1 condition (supplementary figure 2(D)). The starting cell number of macrophages seeded in each tissue was 2.1 × 10^4^ cells, and the cell number of live^+^CD45^+^CD11b^+^ macrophages at ALI day 8 was between 3000–6000 cells across the samples. Considering that the tissue digestion process may lead to some cell death, these data implicate an overall decrease in live^+^CD45^+^CD11b^+^ macrophages at the end of the tissue culture regardless of the presence of other cell types such as dermal fibroblast or keratinocytes.

### 3.2. Macrophage plasticity within Mϕ-FTS model

**Figure 2.**
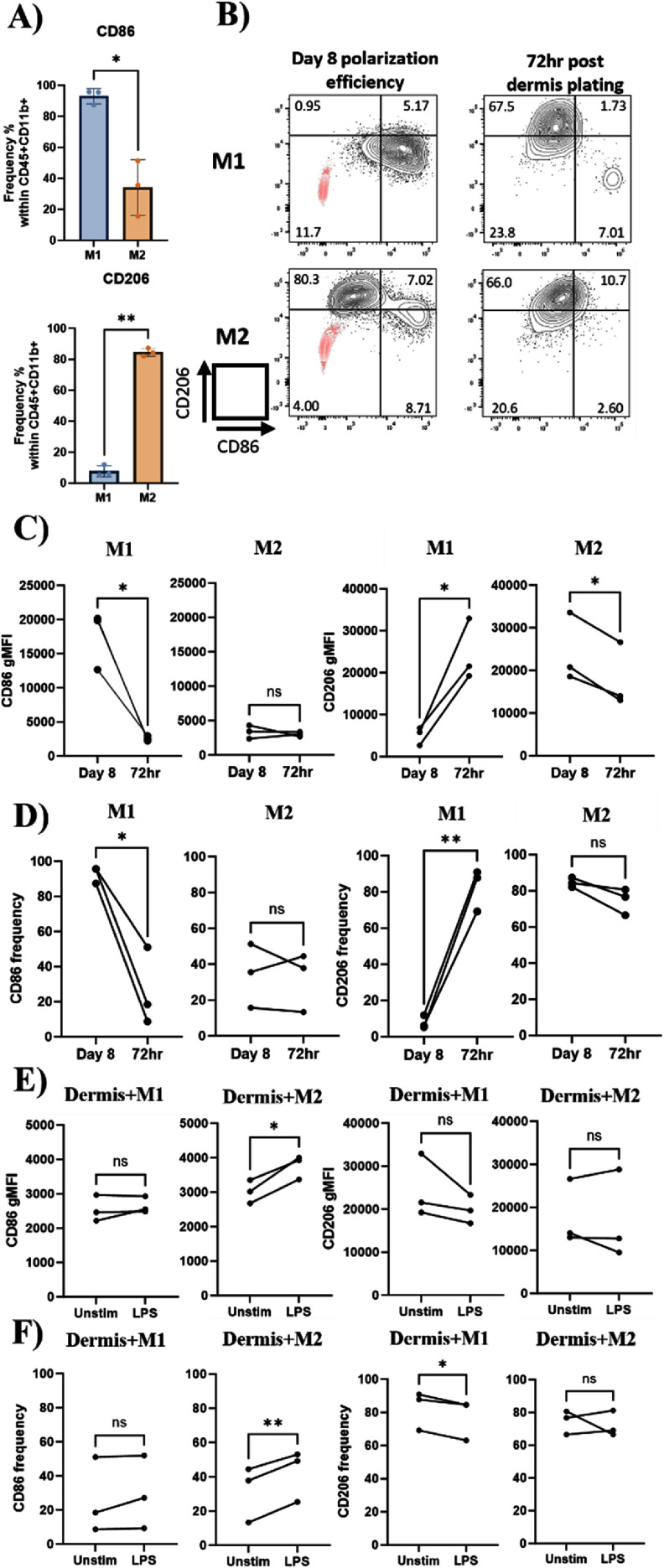
Macrophage plasticity in the dermis under homeostatic and inflammatory stimuli. (A) Frequency of CD86 and CD206 within CD45^+^CD11b^+^ population after M1 or M2 polarization from primary CD14^+^ monocytes for 8 d. On day 8, the macrophages are harvested to mix with dermal fibroblast to generate the dermis (FTS protocol day 0). Harvested M1 and M2 are stained with CD45, CD11b, CD86 and CD206 for polarization efficiency prior to dermis incorporation, *N* = 3 primary monocyte donors. (B) Representative flow plots for day 8 polarization efficiency and single cell suspension from tissue digestion of the dermis after 72 h in culture. Red population in the lower left quadrant of day 8 polarization efficiency plots demonstrate isotype control staining. (C) Geometric mean fluorescent intensity (gMFI) and (D) frequency of CD86 and CD206 expression by macrophages comparing day 8 polarization efficiency prior to mixing with dermal fibroblast to construct the dermis (day 8) and 72 h post dermis plating (72 h). (E) gMFI and (F) frequency of CD86 and CD206 after the tissues were either unstimulated (unstim) or stimulated with 10 *μ*g ml^−1^ LPS for 24 h prior to digestion. All analysis here were gated within the live cell, CD45^+^ (pan-immune cell marker) to eliminate fibroblast within the analysis for tissue digestion samples, CD11b^+^ (pan-myeloid marker) population and plot for CD86 and CD206 (M1 and M2 marker respectively). Each line represents one donor, *N* = 3 primary monocyte donor, *p < 0.05, **p < 0.01, ns: not significant, paired t tests.

Macrophages are known to have high plasticity and the phenotype of polarized M1 and M2 macrophages can be reversed *in vitro* and *in vivo* [44–46]. Prior to incorporating the macrophages into the fibrin-gel with dermal fibroblast, we assessed the polarization efficiency of M1 and M2 macrophages with CD86 and CD206 expression, respectively (Herein referred to as day 8 polarization efficiency in figures). The primary monocytes that underwent M1 polarization generated a higher frequency of CD86^+^ cells (M1: 92.9 ± 4.8%, M2: 34.2 ± 17.8%), and the primary monocytes that underwent M2 polarization generated higher frequency of CD206^+^ cells (M1: 7.8 ± 3.6%, M2:84.6 ± 2.6%. Figures 2(A) and (B): day 8 polarization efficiency). We consistently observed a small population of CD86^+^CD206^−^ as well as CD86^+^CD206^+^ population following M2 polarization (figure 2(B): day 8 polarization efficiency). We also assessed other reported M1 (CD80, HLA-DR) and M2 (CD209, CD163) markers on our polarized macrophages to validate M1 and M2 polarization. We demonstrated that M1 expressed significantly higher CD86 (M1: 40 819 ± 7041.2, M2: 16 125.7 ± 3529), HLA-DR (M1: 54 475.7 ± 8201.5, M2: 29 706.3 ± 3184.3) compared to polarized M2 as reflected by geometric mean fluorescent intensity (gMFI). The polarized M1 expressed higher CD80 expression than M2 but was not significant (M1: 8989.7 ± 2066.4, M2: 7940.7 ± 4280.4). Polarized M2 also express significantly higher CD206 (M1: 4169.3 ± 2139.9, M2: 18 507.3 ± 6640.2), CD209 (M1: 452.8 ± 319.1, M2: 6863 ± 2556.3) compared to M1. M2 expressed higher CD163 compared to M1 but was not significant (M1: 95.55 ± 253.8, M2: 1638.5 ± 596.1, supplementary figure 3).

To determine if the macrophages exhibit plasticity in our skin model, we conducted tissue digestions to obtain single cell suspension for flow cytometry analysis. We harvested dermis + M1/M2 at 72 h post-dermis plating and compared dermis-incorporated macrophage phenotype to their respective day 8 polarization efficiency phenotype by looking at the changes in CD86 and CD206 expressions. It showed that most of the M1 macrophages significantly decreased CD86 expression (from 17 559 ± 4242.1 to 2546.3 ± 381.6, gMFI) and increased CD206 expression (from 5094.7 ± 2131.5 to 24 578 ± 7316.9 gMFI) and frequency (from 7.8 ± 3.6% to 82.6 ± 11.7%), suggesting an early shift into a M2 phenotype (figures 2(C) and (D)). While the M2 macrophages showed statistical significance decrease of CD206 expression with gMFI (from 24 325.3 ± 8103.3 to 17 887.7 ± 7574.1), the frequency of M2 macrophages expressing CD206 remained the same (figure 2(D)). Conversely, when we investigated macrophage plasticity after 72 h in the dermal compartment with and without dermal fibroblast (macrophage alone in hydrogel), we did not see the upregulation of CD206 expression in M1 macrophages (supplementary figure 4). This data demonstrate that M1 macrophage exhibited plasticity as early as 3 d in the dermis in the presence of dermal fibroblast.

The bacterial endotoxin LPS is a well-known innate immune activator leading to cytokine production and inflammation. To examine the stability of our macrophage phenotype in the dermis, we treated the culture with 10 *μ*g ml^−1^ LPS in the basal media for 24 h prior to tissue digestion and analyzed CD86 and CD206 expression of the incorporated M1 and M2 macrophages in the dermis. We harvested the dermis + M1/M2 at 72 h post-dermis plating and compared incorporated macrophages with and without LPS stimulation. We found that incorporated M2 demonstrated plasticity when they were stimulated by LPS reflected by significant increase in CD86 expression (gMFI: 3767.7 ± 343.1) and frequency (CD86^+^ frequency: 42.5 ± 15%) compared to unstimulated condition (unstim gMFI: 3015.7 ± 337.5, CD86^+^ frequency: 31.8 ± 16.4%, figures 2(E) and (F)). Furthermore, LPS stimulation also significantly decreases the frequency of CD206^+^ cells (unstim: 82.6 ± 11.7%, LPS: 77.4 ± 12.4%) in the dermis + M1, while demonstrating a decreasing trend in CD206 expression (figures 2(E) and (F)).

We also subjected the dermis + M1/M2 with a different innate immune activator, synthetic RNA polyinosinic:polycytidylic acid (Poly I:C) to mimic viral infection. When we assessed if 10 *μ*g ml^−1^ Poly I:C treatment would repolarize M2 into M1-like phenotype, we did not observe a significant change in CD86 expression or frequency by incorporated M2. Conversely, Poly I:C treatment significantly decreases CD206 expression by incorporated M1 (unstim gMFI: 24 578 ± 7316.9, Poly I:C gMFI: 19 905.7 ± 5513.4, supplementary figure 5(A)), although not onto original level post M1 polarization (M1 CD206 gMFI: 5094.7 ± 2131.5, figure 2(C): M1 Day 8 polarization efficiency). Although it needs further studies, these results provide strong evidence that the M*ϕ*-FTS model mimics dynamic microenvironment of human skin physiology with timely responses of macrophage plasticity.

### 3.3. Immunocompetence of Mϕ-FTS under inflammatory stimulation

To test if our M*ϕ*-FTS demonstrate immunocompetence, we next tested the effects of treating FTS and M*ϕ*-FTS or dermis and M*ϕ*-dermis with 10 *μ*g ml^−1^ LPS from the basal side of the dermis at different time points of the FTS culture and measure cytokine and chemokine secretions. The supernatants were collected at these time points: 72 h post dermis plating, ALID1 and ALID8, after 24 h of LPS stimulation. 72 h timepoint reflect only dermis or dermis + macrophages while ALID1 and ALID8 reflect FTS or FTS + macrophages.

**Figure 3.**
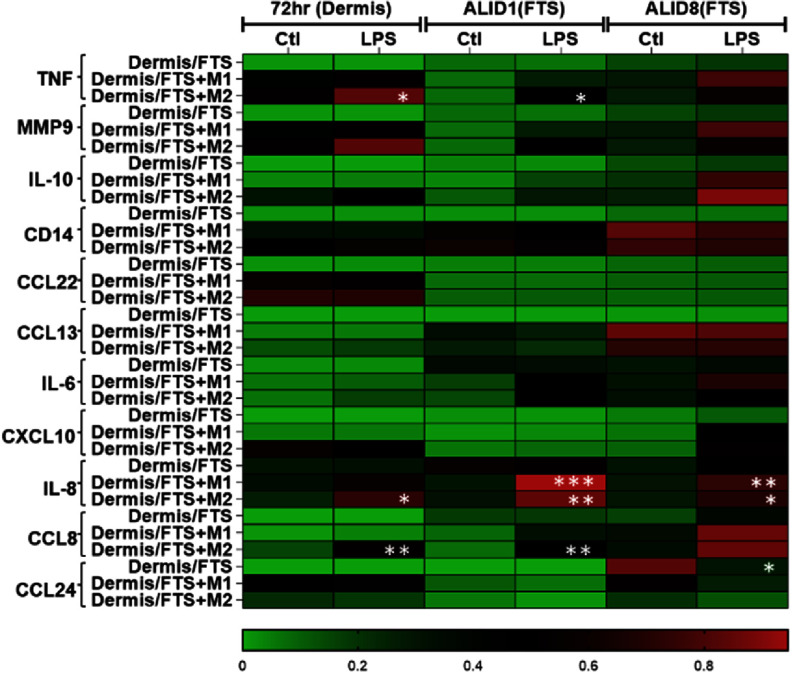
Heatmap reflecting mean values of normalized cytokine level from dermis/FTS, dermis/FTS + M1, and dermis/FTS + M2 at different timepoints in response to LPS. The tissues were stimulated from the basal side with 10 *μ*g ml^−1^ LPS for 24 h prior to the indicated timepoints (72 h post dermis plating, ALID1, ALID8) for Luminex analysis. 72 h timepoint reflect only dermis or dermis + macrophages while ALID1 and ALID8 reflect FTS or FTS + macrophages in the presence of fibroblast, keratinocytes and macrophages. In each experiment, the average raw value of each condition is first obtained, *N* = 2–3 wells per condition. The normalization was conducted to the highest amount detected in each cytokine, across sample type (dermis, FTS, dermis + M1, FTS + M1 and dermis/FTS + M2), across the three timepoints and stimulation condition. The heatmap is generated based on mean normalization values from 4 primary monocyte donors. Statistical analysis was conducted comparing control (ctl) vs LPS using average raw concentration values (across 2–3 replicate wells) within each cytokine and timepoint for *N* = 4 primary monocyte donors (supplementary figure 6). 2way ANOVA, Sidak’s multiple comparisons test, *p < 0.05, **p < 0.01, ***p < 0.001.

First, we analyzed the effects of LPS treatment on our tissues compared to their respective unstimulated samples (control). LPS treatment significantly increased TNF, IL-8 and CCL8 production at early time point (72 h) by dermis + M2 compared to control unstimulated dermis + M2. The LPS treatment significantly decreased CCL24 secretion by FTS (ALID8) compared to unstimulated control FTS, significantly induced IL-8 secretion by FTS + M1 (ALID1, D8) compared to unstimulated control FTS + M1 and significantly induced TNF (ALID1), IL-8 (ALID1, D8), and CCL8 (ALID1) by FTS + M2 compared to control unstimulated FTS + M2 (figure 3, supplementary figure 6).

We next analyzed the effects of macrophage incorporation with LPS stimulation by comparing dermis + M1/M2 to dermis and FTS + M1/M2 with FTS. After LPS stimulation, dermis + M1 secreted significantly higher TNF, and MMP9 at 72 h timepoint compared to dermis alone. FTS + M1 secreted significantly higher MMP9 (ALID1), CD14 (ALID1 and ALID8), CCL13 (ALID1 and ALID8) and IL-8 (ALID1) compared to FTS alone. Dermis + M2 secreted significantly higher TNF, MMP9, IL-10, CD14, CCL13, IL-6, IL-8 and CCL8 compared to dermis alone after LPS stimulation at 72 h timepoint. FTS + M2 secreted significantly higher TNF (ALID1), MMP9 (ALID1, ALID8), IL-10 (ALID1), CD14 (ALID1 and ALID8), CCL13 (ALID1 and ALID8), CXCL10 (ALID1), and CCL8 (ALID1) compared to FTS alone after LPS stimulation. There are also significant cytokine production differences between M1 and M2 incorporation after LPS stimulation. At 72 h timepoint, dermis + M2 has significantly higher production of TNF, MMP9, IL-10, CCL13, and CCL8 compared to dermis + M1 after LPS stimulation. FTS + M2 also has significantly higher levels of CXCL10 production after LPS stimulation compared to FTS + M1 at ALID1 (supplementary figure 7). We also subjected our M*ϕ*-FTS with Poly I:C to mimic viral infection. Addition of 10 *μ*g ml^−1^ Poly I:C in the basal media for 24 h at different timepoints significantly induced IL-6 (ALID1, D8), IL-8 (ALID8) and CCL8 (ALID1, D8) secretion by FTS while there was no significant induction or reduction in M*ϕ*-FTS (supplementary figure 8).

We next analyzed the effects of macrophage incorporation in dermis/FTS on cytokine production without any stimulation. Dermis + M1 has significantly higher MMP9, compared to dermis without any stimulation. FTS + M1 has significantly higher MMP9 (ALID1), CD14 (ALID1, ALID8), CCL13 (ALID1, ALID8) compared to FTS. Dermis + M2 has significantly higher TNF, MMP9, CD14, CCL13 and CXCL10 compared to dermis without stimulation at 72 h timepoint. FTS + M2 has significantly higher MMP9 (ALID1, ALID8), CD14 (ALID1, ALID8), CCL13 (ALID1, ALID8), and lower CCL24 (ALID8) compared to FTS without any stimulation (supplementary figure 7).

### 3.4. Fibrosis induction in Mϕ-FTS

**Figure 4.**
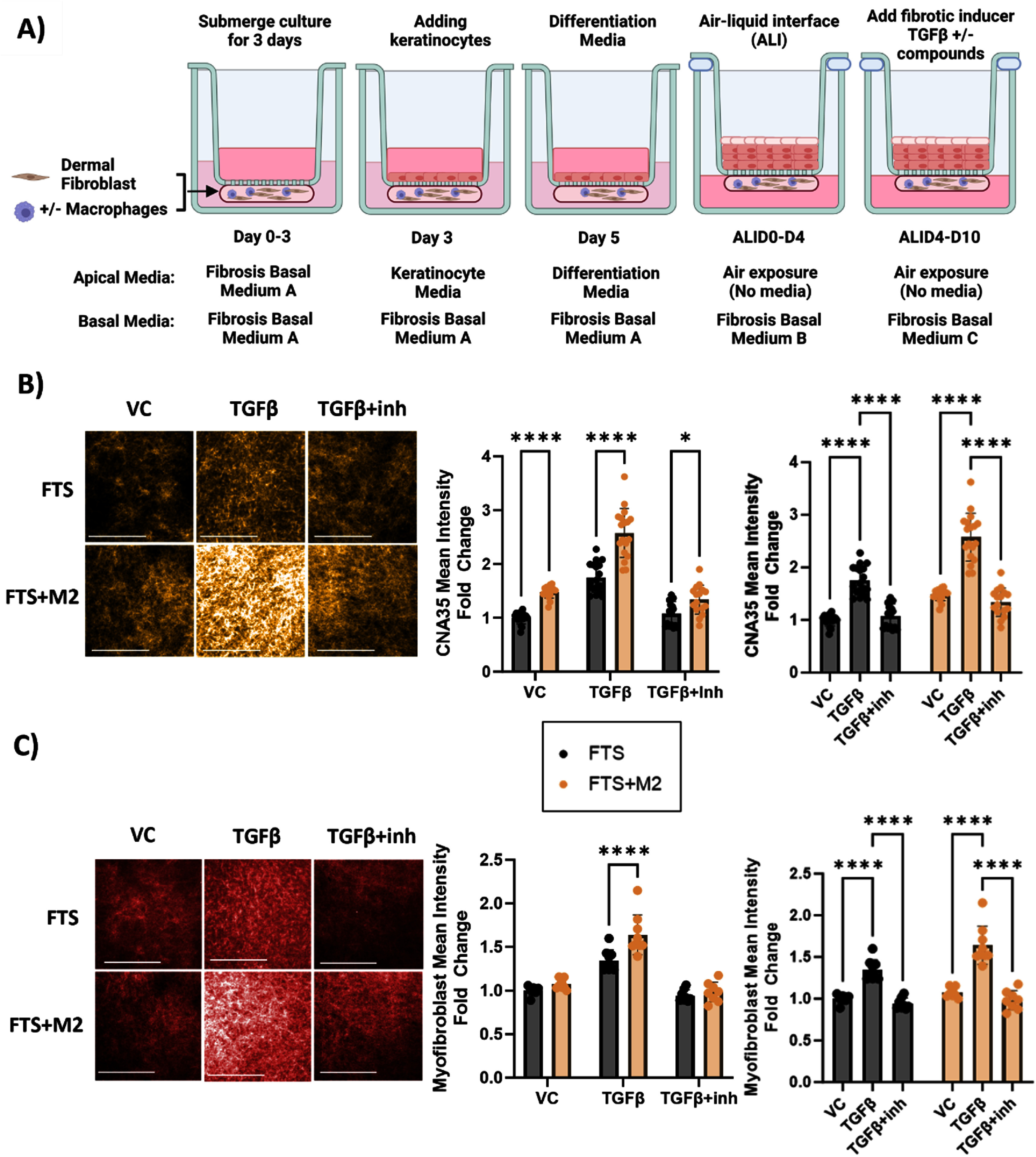
FTS fibrosis model ± M2 macrophages. (A) Schematics depict introducing M2 macrophages to FTS along with the tissue culture and treatment regimen. (B) Representative maximum projection images of mCherry-CNA35-stained FTS and FTS + M2 in vehicle control (VC, DMSO), 10 ng ml^−1^ TGF*β*1 and 10 ng ml^−1^ TGF*β*1 + 10 *μ*M Galunisertib (inh) treatment and mean intensity fold change plots of CNA-35 staining. The graph plots represent *N* = 16–18 wells per condition pooled from 3 primary monocyte donors. (C) Representative maximum projection images of myofibroblast marker-stained FTS and FTS + M2 in VC, 10 ng ml^−1^ TGF*β*1 and 10 ng ml^−1^ TGF*β*1 + inh (10 *μ*M Galunisertib) treatment and mean intensity fold change plots of myofibroblast staining. The graph plot represents *N* = 8–9 wells per condition pooled from 2 primary monocyte donors. The images were collected using a high content imager at 5X magnification, scale bar: 500 *μ*m. Mean intensities were collected using Columbus software. Fold change was calculated within each experiment by normalizing to FTS-VC conditions. *p < 0.05, ****p < 0.0001, 2way ANOVA, Sidak’s multiple comparisons test and Tukey’s multiple comparisons test. Schematics created with BioRender.com.

Macrophages are a highly heterogenous group of innate immune cells. Dysfunction of M1 can promote chronic inflammation, while aberrant activation of M2 promotes development of fibrosis [47]. TGF-*β* is a predominant pathogenic factor for fibrosis induction. We treated M*ϕ*-FTS with TGF*β*1 from the basal side of dermis for 6 d (ALID4–D10; figure 4(A)) to create a model of skin fibrosis. We first conducted a dose response to TGF*β*1 (2.5, 5, and 10 ng ml^−1^) in FTS and FTS + M2 and stained for collagen deposition using mCherry-CNA-35 (CNA35), a collagen binding protein [48]. We detected high mCherry fluorescence signal for either FTS or FTS + M2 at 2.5 ng ml^−1^ TGF*β*1 (supplementary figure 9(A)), which did not increase with higher doses of TGF*β*1, indicating that the media conditions might be pro-fibrotic. FDM media formulations contain higher levels of serum (5% FBS) and a variety of growth factors (supplementary table 1). We then switched to simpler media formulation with lower serum concentration (from 5% to 1.5% FBS) in DMEM without addition of any growth factors (supplementary table 2). We were able to detect a TGF*β*1 dose-dependent collagen deposition and further observed significant potentiation of TGF*β*1-induced collagen production in FTS + M2 at 10 ng ml^−1^ concentration (supplementary figure 9(B)). When we analyzed cytokine production in conditioned media by the two different media conditions, we found that the original high serum FDM media induced high level of fibrosis associated factors and cytokines such as fibronectin and PDGFcc, even without TGF*β*1 treatment. For the high serum FDM media conditions, treatment with 10 ng ml^−1^ TGF*β*1 induced significantly higher levels of fibronectin, pro-collagen I*α*1 and PDGFcc compared to low serum DMEM media conditions. On the other hand, the low serum DMEM media conditions produced higher levels of IL-6, in both vehicle and 10 ng ml^−1^ TGF*β*1 compared to the high serum FDM media (supplementary figure 9(C)). While further investigation remains to be done to elucidate the underlying mechanisms, the low serum DMEM media formulation (known henceforth as fibrosis basal medium) was selected to capture immunocompetence of FTS + M2 for fibrosis induction. Across three different primary monocyte donors, 10 ng ml^−1^ TGF*β*1 consistently increased collagen deposition as reflected by the increase in CNA35 mCherry mean fluorescence intensity compared to the vehicle control (VC): 1.7-fold increase in FTS, 2.6-fold increase in FTS + M2. This induction was prevented by TGF*β* receptor I inhibitor, Galunisertib (10 *μ*M). Furthermore, FTS + M2 significantly increased collagen production in all conditions compared to FTS, even in the absence of TGF*β*1 (figure 4(B)). We further confirmed that both FTS and FTS + M2 showed strong expression of a myofibroblast marker under TGF*β*1 treatment, and it was reversed in the presence of Galunisertib (figure 4(C)). Although FTS + M2 produced significantly higher collagen production than FTS in VC, there was no significant changes in myofibroblast marker expression upon the addition of M2 to the FTS in VC. In contrast, TGF*β*1 treatment significantly increased myofibroblast expression levels more in FTS + M2 than in FTS. This demonstrated that M2 incorporation significantly potentiated TGF*β*-induced fibrosis in FTS.

### 3.5. Fibrosis associated cytokine and factors in Mϕ-FTS skin fibrosis model

**Figure 5.**
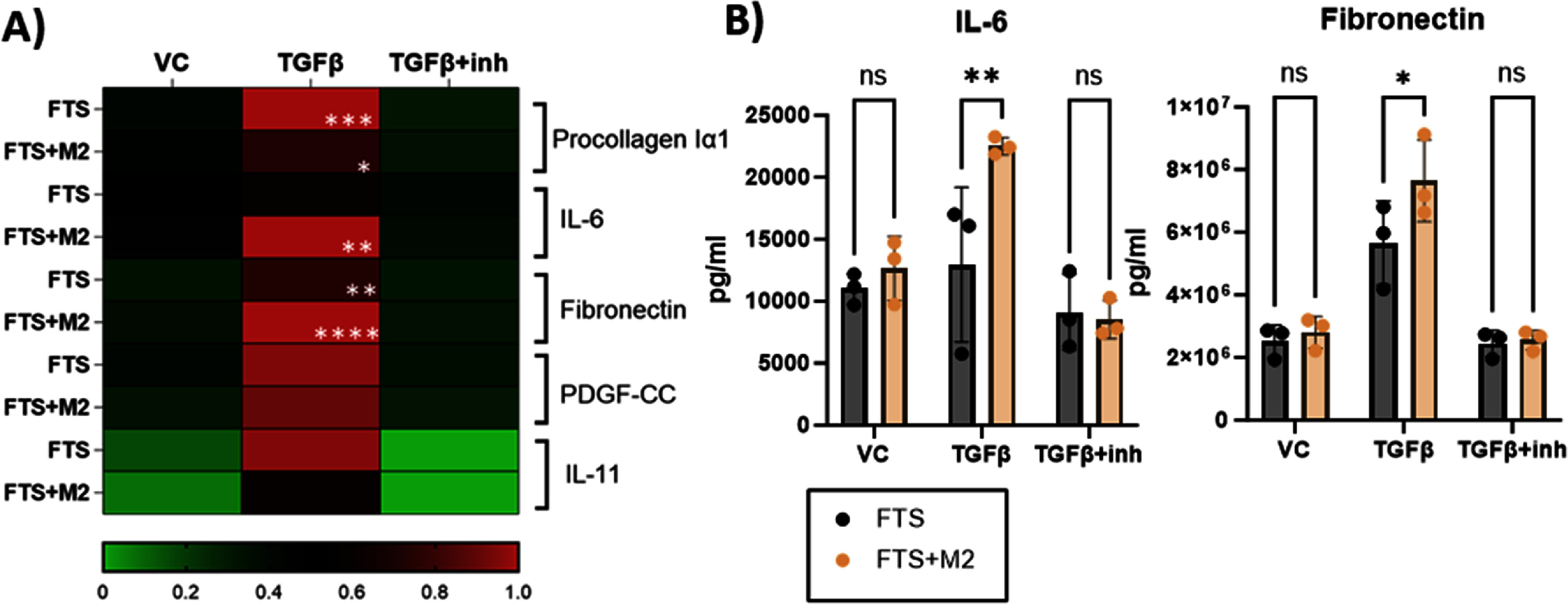
Fibrosis associated cytokine and factors in FTS and FTS + M2 skin fibrosis model. (A) Heatmap reflecting mean normalized values across 3 primary monocyte donors for vehicle control (VC), 10 ng ml^−1^ TGF*β*1 and 10 ng ml^−1^ TGF*β*1 + 10 *μ*M Galunisertib (inh) treatment. In each experiment, the average raw value of each condition is first obtained, *N* = 3 wells per condition. The normalization was conducted to the highest amount detected in each cytokine, across FTS and FTS + M2, and across treatment conditions. Statistical analysis was conducted comparing VC, TGF*β*1 and TGF*β*1 + inh using raw concentration values (average of 3 replicates) of each cytokine across *N* = 3 primary monocyte donors (supplementary figure 10). The statistics on the heatmap only showed statistics of VC vs TGF*β*1. Conditioned media were collected at ALID10 for Luminex analysis. (B) IL-6 and fibronectin concentration level, average values across 3 replicates per condition from *N* = 3 primary monocyte donors. 2way ANOVA, Tukey’s and Sidak’s multiple comparisons test, *p < 0.05, **p < 0.01, ***p < 0.001, ****p < 0.0001.

We also analyzed key fibrosis associated cytokines and factors in the conditioned media from ALID10. 10 ng ml^−1^ TGF*β*1 treatment significantly induced pro-collagen I*α*1 and fibronectin secretion in both FTS and FTS + M2 (figure 5(A)). The TGF*β*1 + Galunisertib (inh, 10 *μ*M) treatment decreases pro-collagen I*α*1 and fibronectin production in both FTS and FTS + M2 (supplementary figure 10). The TGF*β*1 treatment significantly induced IL-6 production only in FTS + M2 conditions, demonstrating a M2 potentiation effect (figures 5(A) and (B)). Furthermore, there was a M2 potentiation in TGF*β*-induced fibronectin production compared to FTS (figure 5(B)).

## 4. Discussion

We have successfully developed a protocol to incorporate macrophages into the FTS model that maintained intact barrier function and epidermis differentiation. LPS stimulation further confirmed that the M*ϕ*-FTS model can produce multiple pro-inflammatory cytokines reminiscent of the innate immunity of the human skin, supporting immunocompetence of the model. Macrophages have been typically categorized into two states, M1 and M2, characterized by different functions, cytokine profiles, transcriptional profiles, and signaling mechanisms that determine their polarization state [49]. M1 is known to produce pro-inflammatory cytokines such as TNF, IL-12, IL-6, IL-1*β* and IL-23, and M2 produce anti-inflammatory or immunoregulatory cytokines such as IL-10 and TGF*β* [50]. Cues from the microenvironment induce macrophages to switch from one functional phenotype to the other. Pre-polarization into M1 phenotype (via LPS and IFN*γ*) was shown to have subsequent enhanced response to M2 stimuli (IL-4 and IL-13) by increasing M2 marker CD206, even higher than previously unpolarized cells. Conversely, the ability of pre-differentiated M2 to acquire M1-like state via LPS and IFN*γ* stimulation requires a higher concentration of stimulus for reprogramming from M2 into M1-like cells [51]. This implies that M1 has higher plasticity than M2 macrophages. In our model, we observed physiologically relevant macrophage plasticity in two settings: (1) without any stimulation, the dermis environment significantly induced M1 to express CD206 while decreasing CD86 expression, aligning with the previous reports of the M2 marker expressions, CD206 and CD163, by dermal tissue-resident macrophages in homeostasis [52, 53]. (2) In the presence of LPS stimulation of dermis + M2, incorporated M2 subsequently upregulate CD86 expression and the production of TNF, aligning with previous reports that LPS and IFN*γ* stimulation of previously polarized M2 macrophages induce the production of TNF and IL-10 as well as CD86 expression [54]. IL-6 is a pleiotropic cytokine and has been shown to enhance development of M2 macrophages by further upregulating CD206 expression [55]. IL-6 promotes M2 polarization by increasing IL-4 receptor *α* expression and IL-6 neutralization blunted *Il4ra* expression [56, 57]. IL-6 can be produced by dermal fibroblast, and we can detect IL-6 in our conditioned media without any LPS stimulation throughout our skin culture (72 h, ALID1, and ALID8, supplementary figure 6). Potentially, IL-6 production by our skin construct promotes CD206 expression by the incorporated M1 macrophages. This supports that the skin microenvironment promotes an M2-like phenotype, mimicking the homeostatic condition.

We still detected viable macrophages by the end of our skin culture (ALI day 8) even though we observed a significant decrease in the frequency of viable macrophages at ALI day 8 compared to earlier time point of the culture (72 h post dermis plating). We also observed that in the absence of dermal fibroblast (macrophage alone in hydrogel), we saw a lower frequency of live macrophages compared to those in culture with dermal fibroblast. This agrees with previous reports that fibroblast can provide macrophage colony-stimulating factor (M-CSF), an essential growth factor that promotes survival, differentiation and proliferation of macrophages [58]. Future studies using a modular approach will explore the role of how different cell types in our FTS can affect macrophage viability in the culture.

M2 macrophage can be further divided into different subsets depending on the external modulators: M2a (induced by IL-4 or IL-13), M2b (induced by immune complexes and TLR agonists) and M2c (induced by glucocorticoids and IL-10). M2a produces high levels of IL-8 [59], fibronectin 1 and matrix associated protein *β*IGH3, which plays a role in fibrogenesis [60], and chemokines such as CCL22 [61], CCL17 [62], CCL2 [59], CCL3 [59], CCL24 [59]. M2b subset express CD86 and produces both anti- and pro-inflammatory cytokines IL-10, IL-6, IL-1*β* and TNF [63, 64]. The M2c subset produces a high amount of IL-10 and TGF*β* and expresses high levels of arginase to promote tissue regeneration and angiogenesis [65, 66]. We consistently observed a population of M2 that also express CD86 using our polarization method (figure 2(B)), suggesting that FTS + M2 contains a population of M2b. The cytokine profile of M*ϕ*-FTS under LPS stimulation demonstrated a variety of cytokine and chemokine production that do not follow the strict M1 and M2 subset categories due to the complexity of FTS. Although it needs further studies, these results provide strong evidence that the M*ϕ*-FTS model mimics dynamic microenvironment of human skin physiology with timely responses of macrophage plasticity.

The FTS model successfully demonstrated fibrosis induction by TGF*β*1 treatment, and the M2 incorporation significantly potentiated the fibrosis induction by TGF*β*1 as reflected in collagen production and myofibroblast marker expression. Interestingly, the addition of M2 to the FTS by itself without any TGF*β*1 treatment significantly induced collagen production while maintaining low levels of myofibroblast transformation (figures 4(B) and (C) vehicle control condition). This suggests that the incorporation of M2 may promote ECM production to support the maturation of FTS, highlighting the intricacy of the wound healing process and fibrosis initiation/progression by its dysregulation. This data also supports the reported role of M2 in tissue remodeling under homeostatic condition or in tissue repair under inflammatory conditions by promoting ECM production [12]. In addition to collagen production, FTS + M2 significantly produced fibronectin and IL-6 under the TGF*β*1 treatment. Macrophages have been shown to produce ECM such as fibronectin and collagen under homeostasis and in the presence of TGF*β*1 stimulation [67–71]. Fibronectin splice variant ED-A and TGF*β*1 is shown to promote myofibroblasts differentiation [72]. Fibronectin polymerization is necessary for collagen matrix deposition and inhibiting fibronectin has been shown to attenuate fibrosis [73–75]. IL-6 is a multifunctional cytokine and is a critical modulator of inflammatory and reparative processes in wound healing. IL-6 also has been shown to promote fibroblast to synthesize collagen and transdifferentiate into myofibroblast [27, 76]. IL-6 also increases fibronectin expression by M2 macrophages [77]. Ablation of dermal macrophages has been shown to compromise wound morphology, delay healing time, reduce neovascularization, and impair wound closure [78]. Other self-assembled skin equivalents and tissue spheroids that model skin fibrosis with varying complexities by incorporating systemic sclerosis (SSc)-derived or healthy human dermal fibroblasts, iPSC-derived fibroblast and some including human dermal microvascular endothelial cells (ECs) and immune cells like mast cells and macrophages have been previously established [79–85]. *Ex vivo* SSc skin transplants to characterize skin fibrosis have also been established [86]. However, our immunocompetent 3D skin model demonstrated the capability of a HT amenable, reproducible and promising platform for HT screening (HTS) of anti-fibrotic drugs using HT readouts of collagen level by CNA-35 staining and cytokine biomarkers such as IL-6 and fibronectin.

Immunocompetent FTS models with increasing tissue complexity to capture more pathophysiological relevant outcomes in skin diseases remain to be developed. In SSc, a systemic autoimmune disease that causes vasculopathy and fibrosis, EC injury, apoptosis, and subsequent EC dysfunction is implicated to be the initiating event of SSc and precedes the development of fibrosis by months or years [87–89]. This vascular repair machinery dysfunction triggers vascular lesions, fibrosis, and a series of inflammatory and pro-fibrotic processes. There is growing evidence that a pathological stimulus, such as TGF*β*, TNF‐*α*, IL‐1, interferon, hypoxia, and ROS may induce ECs transdifferentiation toward myofibroblasts, through the endothelial‐to‐mesenchymal transition [88]. These key features remain a limitation of the model due to the lack of vasculature. Future work using protocols established in our laboratory will explore the role of vasculature in fibrosis formation in FTS and M*ϕ*-FTS.

## 5. Conclusion

We generated macrophage-incorporated FTS tissue equivalents in a HT amenable format. Our M*ϕ*-FTS model is capable in generating an immune response and demonstrate physiological relevant macrophage plasticity in response to the tissue microenvironment. The fibrosis model using the immunocompetent M*ϕ*-FTS with TGF*β*1 stimuli provides extensive opportunities for studying underlying mechanism of macrophage-related disease initiation and progression. We believe that this human skin fibrosis model using CNA-35 labeling as a real time measure of collagen deposition holds a promise for HTS of anti-fibrotic drugs.

## Data Availability

The data cannot be made publicly available upon publication because they are not available in a format that is sufficiently accessible or reusable by other researchers. The data that support the findings of this study are available upon reasonable request from the authors.
